# African swine fever virus MGF505–6R attenuates type I interferon production by targeting STING for degradation

**DOI:** 10.3389/fimmu.2024.1380220

**Published:** 2024-05-10

**Authors:** Manman Yao, Hua Cao, Wentao Li, Zihui Hu, Zhenxiang Rong, Mengge Yin, Linxing Tian, Dayue Hu, Xiangmin Li, Ping Qian

**Affiliations:** ^1^ National Key Laboratory of Agricultural Microbiology, Hubei Hongshan Laboratory, Huazhong Agricultural University, Wuhan, Hubei, China; ^2^ College of Veterinary Medicine, Huazhong Agricultural University, Wuhan, Hubei, China; ^3^ Key Laboratory of Preventive Veterinary Medicine in Hubei Province, The Cooperative Innovation Center for Sustainable Pig Production, Wuhan, Hubei, China

**Keywords:** African swine fever virus, MGF505–6R, STING, type I interferon, immune evasion

## Abstract

African swine fever (ASF) is an acute hemorrhagic and devastating infectious disease affecting domestic pigs and wild boars. It is caused by the African swine fever virus (ASFV), which is characterized by genetic diversity and sophisticated immune evasion strategies. To facilitate infection, ASFV encodes multiple proteins to antagonize host innate immune responses, thereby contributing to viral virulence and pathogenicity. The molecular mechanisms employed by ASFV-encoded proteins to modulate host antiviral responses have not been comprehensively elucidated. In this study, it was observed that the ASFV MGF505–6R protein, a member of the multigene family 505 (MGF505), effectively suppressed the activation of the interferon-beta (IFN-β) promoter, leading to reduced mRNA levels of antiviral genes. Additional evidence has revealed that MGF505–6R antagonizes the cGAS-STING signaling pathway by interacting with the stimulator of interferon genes (STING) for degradation in the autophagy-lysosomal pathway. The domain mapping revealed that the N-terminal region (1–260aa) of MGF505–6R is the primary domain responsible for interacting with STING, while the CTT domain of STING is crucial for its interaction with MGF505–6R. Furthermore, MGF505–6R also inhibits the activation of STING by reducing the K63-linked polyubiquitination of STING, leading to the disruption of STING oligomerization and TANK binding kinase 1 (TBK1) recruitment, thereby impairing the phosphorylation and nuclear translocation of interferon regulatory factor 3 (IRF3). Collectively, our study elucidates a novel strategy developed by ASFV MGF505–6R to counteract host innate immune responses. This discovery may offer valuable insights for further exploration of ASFV immune evasion mechanisms and antiviral strategies.

## 1 Introduction

African swine fever (ASF) is a highly contagious and lethal disease of pigs caused by the African swine fever virus (ASFV). It was first reported in Kenya during the 1920s and has since rapidly spread in Africa and Eurasia (1, 2). The first outbreak in China was documented in August 2018, causing severe economic and ecological burdens to the pig industry (3). ASFV primarily targets monocyte-macrophages for infection. However, the mechanisms of ASFV infection are still not fully understood (4). The clinical symptoms associated with the virulent strains include multi-organ hemorrhages, hyperthermia, ataxia, and immunosuppression, with a high mortality rate approaching 100% (5–7). Until now, there have been no effective vaccines or available therapies for ASF due to its genetic complexity and sophisticated immune evasion strategies. The strict culling of infected pigs tends to be the only effective method to control the rapid spread of ASFV (8–10).

Being the only member of the family Asfarviridae, the genus Asfavirus, and the only known DNA arbovirus, ASFV has been classified into 24 genotypes based on the gene sequence of the major capsid protein p72 (2, 11, 12). ASFV is a large, icosahedral, double-stranded DNA (dsDNA) virus with a linear genome ranging from 170–193 kb in length. It contains 150–167 open reading frames (ORFs) that encode 150–200 proteins, depending on the virus strain. These proteins include 68 structural proteins and over 100 nonstructural proteins that contribute to virus replication, assembly, virulence, and host immune defense. However, a full understanding of these proteins has not yet been achieved (13, 14). The identification and characterization of the proteins responsible for innate immune evasion will contribute to future research on antiviral strategies against ASFV (15).

Innate immune responses serve as the host’s first line of defense when encountering invading pathogenic agents (16). Cyclic GMP-AMP synthase (cGAS) is a pattern recognition receptor that is universally distributed in various cells and primarily responsible for detecting cytosolic dsDNA. Upon invasion by a dsDNA virus, cGAS first recognizes the cytosolic viral dsDNA and then catalyzes the synthesis of the second messenger molecule 2’ 3’-cGAMP to initiate signaling cascades. The molecule subsequently binds to and activates the endoplasmic reticulum (ER)-anchored adapter of the stimulator of interferon genes (STING), leading to the oligomerization and translocation of STING. Subsequently, the downstream signaling molecule TANK-binding kinase 1 (TBK1) is recruited and activated. This process leads to the phosphorylation of the transcription factor interferon regulatory factor 3 (IRF3), resulting in the formation of dimers that then migrate to the nucleus. There, they initiate the transcription of type I interferon and downstream antiviral genes (17–19).

To facilitate infection and replication, ASFV has evolved multiple immune evasion strategies to counteract host immune responses (20–22). Previous studies have revealed that the multigene family 505 (MGF505) and MGF360 genes inhibit the production of type I interferon. Enhanced innate immune responses were elicited upon the deletion of specific genes compared to the parental strains (15, 23). It has been reported that ASFV blocks the production of IFN-β by negatively regulating the cGAS-STING pathway during infection (24). Specifically, ASFV MGF505–7R promotes the expression of the autophagy-related protein ULK1 to degrade STING, consequently inhibiting the production of IFN-β (25). More recently, it has been proven that ASFV MGF505–7R also inhibits the Janus-activated kinase-signal transducer and activator of transcription (JAK-STAT) signaling pathway by interacting with interferon regulatory factor 9 (IRF9) and blocking the nuclear translocation of IFN-stimulated gene factor 3 (ISGF3) (26). Furthermore, ASFV MGF505–11R has been shown to antagonize cGAS-STING-mediated IFN-β production by degrading the STING protein through the lysosomal, ubiquitin-proteasome, and autophagy pathways (27).

To further explore the immune evasion mechanisms utilized by ASFV-encoded proteins, we screened 29 viral proteins belonging to the MGF family using dual-luciferase reporter assays. Among these, MGF505–6R exhibited significant antagonistic effects on Sendai virus (SeV)-induced activation of the IFN-β promoter. Our study revealed that the MGF505–6R protein dose-dependently decreased the activation of the IFN-β promoter triggered by poly(dA:dT) and cGAS-STING. Furthermore, MGF505–6R impaired the cGAS-STING signaling pathway by interacting with STING for degradation through the autophagy-lysosome pathway. Specifically, the N-terminal of MGF505–6R was essential for the interaction with STING, and the CTT domain of STING was crucial for interacting with MGF505–6R. Moreover, MGF505–6R impaired the activation of STING by facilitating the removal of K63-linked polyubiquitination from STING, thereby disrupting the phosphorylation and nuclear translocation of IRF3. Collectively, our findings have revealed a previously unrecognized strategy employed by MGF505–6R in manipulating innate immune responses. This discovery of MGF505–6R provides insights into how the African swine fever virus manipulates innate immunity.

## 2 Materials and methods

### 2.1 Cells and viruses

HEK-293T (ATCC CRL-11268), SK6 (Wuhan University), and HeLa (ATCC CCL-2) cells were cultured in Dulbecco’s Modified Eagle Medium (DMEM, Cytiva, Marlborough, MA, USA) supplemented with 10% fetal bovine serum (FBS, Gibco, USA) and 1% penicillin-streptomycin (Gen-View, Jacksonville, FL). The primary porcine alveolar macrophages (PAMs) were isolated from 20- to 30-day-old piglets and cultured in RPMI 1640 medium (Gibco, Waltham, MA, USA) supplemented with 10% FBS. All cells were maintained at 37°C, containing 5% CO_2_. ASFV strain SXH1 of genotype II was isolated from clinical samples using PAM cells as previously described (28). The enhanced green fluorescent protein-tagged vesicular stomatitis virus (eGFP-VSV) and Sendai virus (SeV) were amplified as previously described and stored at -80°C (29).

### 2.2 Plasmids

The full-length MGF505–6R gene was amplified based on the ASFV strain Wuhan 2019–1 (GenBank accession No. MN393476) and tagged with 6His through cloning into the pTRIP-CMV vector. The truncations of MGF505–6R-N (1–260aa), MGF505–6R-C (261–526aa), STING-TM (1–154aa), STING-NT (1–180aa), STINGΔCTT (1–340aa), and STINGΔTM (154–379aa) were subcloned into the pTRIP-CMV vector with a Flag tag. The plasmids for human Flag-cGAS, Flag-STING, HA-STING, Flag-TBK1, Flag-IKKϵ, and Flag-IRF3 were cloned into the pTRIP-CMV or pcDNA3.1-HA vector. Flag-MAVS and Flag-TRIF were kindly provided by Meilin Jin (Hua Zhong Agricultural University). Porcine cGAS and STING sequences were cloned into the pTRIP-CMV vector to yield Flag-cGAS and Flag-STING expression plasmids. pRL-TK and pGL3-IFN-β-Luc plasmids were used for dual luciferase assays (29). The HA-tagged ubiquitin and ubiquitin mutants were cloned into the pcDNA3.1-HA vector using overlap PCR or mutagenesis kits (Vazyme, China).

### 2.3 Antibodies and reagents

Mouse monoclonal antibodies against His-tag (66005–1-Ig), HA-tag (66006–2-Ig), and GAPDH (60004–1-Ig), as well as rabbit polyclonal antibodies against Flag-tag (20543–1-AP), cGAS (26416–1-AP), STING (19851–1-AP), TBK1 (28397–1-AP), IRF3 (11312–1-AP), Phospho-IRF3 (29528–1-AP), ATG7 (10088–2-AP), and LC3B (18725–1-AP) were procured from Proteintech (Rosemont, IL, USA). Rabbit His-tag (PM032) polyclonal antibodies, mouse Flag-tag monoclonal antibody (M185–3L), goat anti-mouse (72–8042-M001), and goat anti-rabbit polyclonal antibodies (72–8067-M001) were purchased from Medical and Biological Laboratories (Nagoya, Japan). A human anti-p54 antibody was prepared as previously described (28). The jetPRIME for transfection was purchased from Polyplus-Transfection (Illkirch, France). Alexa Fluor 555-labeled goat anti-mouse antibody (A-21424), Alexa Fluor 488-labeled goat anti-rabbit antibody (A32731TR), and 4’,6-diamidino-2-phenylindole (DAPI, 62247) were purchased from Invitrogen (Carlsbad, CA, USA). Caspase inhibitor Z-VAD-FMK (HY-16658B) and autophagosome inhibitor chloroquine (CQ, HY-17589A) were procured from MedChemExpress (NJ, USA). Hieff^®^ qPCR SYBR Green Master Mix (Low Rox Plus) (11202ES03) and Hifair^®^V one-step RT-gDNA digestion SuperMix (11142ES10) for qPCR were purchased from YEASEN (Shanghai, China). The proteasome inhibitor MG132, dimethyl sulfoxide (DMSO), firefly luciferase reporter gene assay kit, poly-L-lysine, protease inhibitor cocktail, and protein A+G agarose gel were purchased from Beyotime Biotechnology (Shanghai, China). The nuclear and cytoplasmic extraction kit was acquired from Thermo Fisher Scientific (MA, USA). Cycloheximide (CHX) was purchased from Sigma-Aldrich (St. Louis, MO, USA).

### 2.4 Dual-luciferase reporter assays

To assess the effects of MGF505–6R overexpression on IFN-β promoter activity. HEK-293T and SK6 cells were co-transfected with His-MGF505–6R, pGL3-IFN-β-Luc, and pRL-TK, along with the indicated signaling molecules for 24 h, or treated with 1 μg/mL of poly(dA:dT) for 12 h. Subsequently, the cells were lysed for firefly and Renilla assays using the dual luciferase reporter gene assay kit, following the manufacturer’s protocol (Beyotime Biotechnology, Shanghai, China).

### 2.5 RNA extraction and quantitative PCR (qPCR)

For the detection of mRNA levels of IFN-β and antiviral genes ISG54, ISG56, CXCL10, and TNF-α, HEK-293T and SK6 cells were transfected with either an empty vector or His-MGF505–6R, along with the indicated signaling molecules for 24 h, or treated with 1 μg/mL poly(dA:dT) for 12 h. For the detection of MGF505–6R transcriptional kinetics, porcine alveolar macrophages (PAMs) infected with 1 MOI of ASFV strain SXH1 were collected at 0 h, 6 h, 12 h, 24 h, and 36 h post-infection. Subsequently, total RNA was extracted using TRIpure reagent (Aidlab Technologies, Beijing, China), and 1 μg of RNA was reverse transcribed into cDNA using Hifair^®^V one-step RT-gDNA digestion SuperMix (YEASEN, Shanghai, China). The cDNA was then subjected to qRT-PCR detection with Hieff^®^ qPCR SYBR Green Master Mix (Low Rox Plus) (YEASEN, Shanghai, China) according to the manufacturer’s protocol. The relative mRNA levels were normalized to GAPDH mRNA, referring to the 2-ΔΔCT method. All primers used for qPCR detection are listed in **Supplementary Table 1** in the **Supplementary Material**.

### 2.6 Co-immunoprecipitation and western blot analysis

For co-immunoprecipitation (co-IP) assays, HEK-293T or SK6 cells were pre-seeded into 6-well plates and cultured until they reached 70–80% confluence. Subsequently, the cells were transfected with the indicated plasmids for 24 h. Afterward, the cells were lysed with 150 μL of NP-40 lysis buffer (50 mM HEPES, pH 7.4, 200 mM NaCl, 1 mM EDTA, 1% NP-40) supplemented with a protease inhibitor cocktail (Beyotime Biotechnology, Shanghai, China) for 40 min on ice. Then, 40 μL of supernatant from lysed cells was directly used for western blot assays with 12% sodium dodecyl sulfate polyacrylamide gel electrophoresis (SDS-PAGE) after being mixed with 5× loading buffer and boiled at 95°C for 10 min. To test for co-immunoprecipitation (co-IP), the remaining 100 μL of supernatant was incubated with the indicated antibodies for 2 h. Then, 20 μL of protein A+G agarose gel (Beyotime Biotechnology, Shanghai, China) was added and incubated for an additional 1 h. Subsequently, the mixtures were thoroughly washed five times to remove nonspecific precipitates. The protein samples were separated using 12% SDS-PAGE and then transferred onto a polyvinylidene difluoride (PVDF) membrane (Sigma-Aldrich, St. Louis, MO, USA). Subsequently, the membrane was blocked with 5% skimmed milk in PBST buffer (PBS containing 0.1% Tween 20) for 1 h at 37°C or overnight at 4°C, and then incubated with the indicated primary antibodies for 1.5 h. After three washes, the membranes were incubated with horseradish peroxidase-coupled goat anti-mouse IgG or goat anti-rabbit IgG (1:5000, MBL, Japan) for an additional 40 min, followed by three washes. Finally, the protein bands were visualized using the ECL chemiluminescence system (Thermo Fisher Scientific, Waltham, USA).

### 2.7 Immunofluorescence assay

For immunofluorescence the coverslips were placed in 24-well plates and treated with poly-L-lysine before seeding HEK-293T cells. Then the indicated plasmids were transfected into a 70–80% confluent monolayer for 24 h. Subsequently, the cells were fixed with 4% formaldehyde for 15 min at 37°C and permeabilized with 0.2% Triton X-100 for 20 min at 4°C. After being washed three times with PBST, the cells were blocked with 1% bovine serum albumin (BSA) for 1 h at 37°C. Following this, the indicated primary antibodies were added and incubated for 1.5 h at 37°C. Subsequently, the samples were washed before adding Alexa Fluor 555 goat anti-mouse and Alexa Fluor 488 goat anti-rabbit antibodies, which were then incubated for 40 min. The nucleus were stained with DAPI for 10 min. After washing, the images were observed and captured using a confocal laser scanning microscope (Ti-U-Nikon, Tokyo, Japan).

### 2.8 RNA interference

Short hairpin RNA (shRNA) targeting STING was cloned into the pLKO.1 vector (pLKO.1-shSTING). To generate STING-knockdown cells, HEK-293T cells were transfected with pLKO.1-shSTING, along with the packaging plasmids psPAX2 and pMD2.G. After 48 h transfection, the recombinant lentiviruses were collected from the supernatant of the cultured cells. Subsequently, HeLa cells were transduced with the collected recombinant lentiviruses in the presence of polybrene, and the knockdown efficiency was assessed using western blot analysis.

The small interfering RNA (siRNA) targeting autophagy-related gene 7 (siRNA-ATG7) was designed and synthesized by Sangon (Shanghai, China). To generate ATG7-knockdown cells, HEK-293T cells were transfected with siRNA-ATG7 for 12 h and subsequently analyzed using western blotting. The sequences of siRNA-ATG7 are provided in **Supplementary Table 2**.

### 2.9 Statistical analysis

The data were analyzed using GraphPad Prism 8.0 software (GraphPad Software Inc., La Jolla, CA, USA) and are presented as the mean ± standard deviation (SD) from at least two independent experiments. Statistical analyses were conducted using an unpaired, two-tailed Student’s t-test, assuming unequal variance (*, p < 0.05; **, p < 0.01; ***, p < 0.001; ns, not significant).

## 3 Results

### 3.1 ASFV MGF505–6R attenuates the cGAS-STING signaling pathway in swine kidney-6 (SK6) and HEK-293T cells

To identify the ASFV-encoded proteins that affect the type I interferon signaling pathway, we screened 29 viral proteins belonging to the MGF family using a dual luciferase reporter system in HEK-293T cells. The results indicated that several proteins from the MGF505 family significantly inhibited the activation of the IFN-β promoter induced by Sendai virus (SeV) (**Supplementary Figure 1**). Among these proteins, MGF505–6R displayed the most pronounced antagonistic effects.

**Figure 1:**
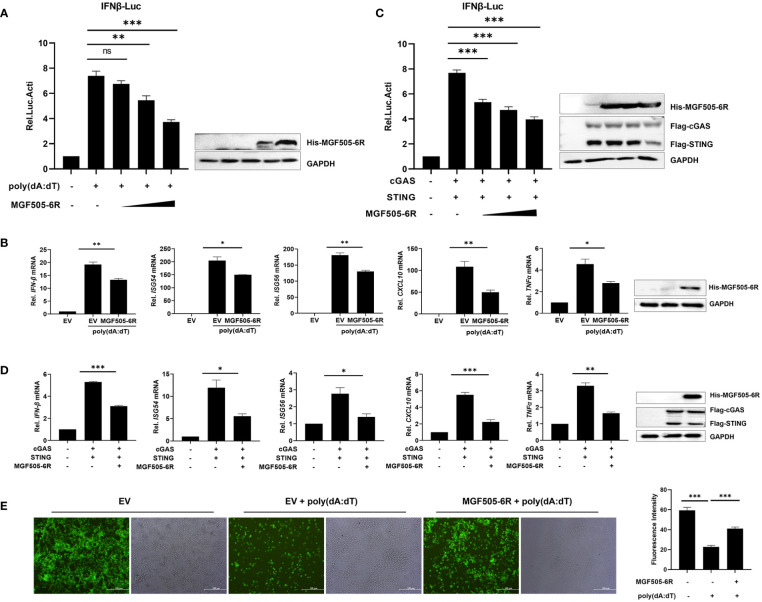
ASFV MGF505–6R attenuates the cGAS-STING signaling pathway in swine kidney-6 (SK6). **(A)** SK6 cells were transfected with pGL3-IFN-β-Luc (0.1 μg), pRL-TK (0.05 μg), and increasing doses of His-MGF505–6R plasmids for 24 h. Following that, 1 μg/mL of poly(dA:dT) was transfected for another 12 h before luciferase assays. **(B)** SK6 cells were transfected with His-MGF505–6R for 24 h and then re-transfected with 1 μg/mL of poly(dA:dT) for 12 h before the detection of IFN-β, ISG54, ISG56, CXCL10, and TNFα mRNA levels. **(C)** SK6 cells were transfected with pGL3-IFN-β-Luc, pRL-TK, Flag-cGAS, and Flag-STING, along with increasing doses of His-MGF505–6R plasmids for 24 h before luciferase assays. **(D)** SK6 cells were transfected with Flag-cGAS, Flag-STING, and His-MGF505–6R for 24 h before the detection of IFN-β, ISG54, ISG56, CXCL10, and TNFα mRNA levels. **(E)** SK6 cells transfected with empty vector (EV) or His-MGF505–6R were re-transfected with poly(dA:dT) for 12 h and then infected with 0.01 multiplicity of infection (MOI) of enhanced green fluorescent protein-tagged vesicular stomatitis virus (eGFP-VSV) for 12 h before observation on a fluorescence microscope. The average fluorescence intensity was analyzed using ImageJ software. The data are shown as the mean ± SD; n = 3. *, p < 0.05; **, p < 0.01; ***, p < 0.001; ns, not significant.

To further dissect the antagonistic mechanisms employed by MGF505–6R, which have not been reported previously, we conducted dual luciferase assays by transfecting pGL3-IFN-β-Luc, pRL-TK, along with His-MGF505–6R or empty vector (EV) plasmids into swine kidney-6 (SK6) cells, and using poly(dA:dT) as an IFN-β inducer. The results indicated that MGF505–6R measurably attenuated poly(dA:dT)-induced IFN-β promoter activation in a dose-dependent manner in SK6 cells (**Figure 1A**). Furthermore, the mRNA levels of IFN-β and antiviral genes were investigated in SK6 cells after transfection with the indicated plasmids and treatment with poly(dA:dT). The results showed that overexpression of MGF505–6R effectively reduced the mRNA levels of IFN-β and the antiviral genes ISG54, ISG56, CXCL10, and TNFα in SK6 cells (**Figure 1B**). To investigate whether MGF505–6R antagonizes type I interferon by targeting the cGAS-STING axis, a general cytosolic dsDNA sensing pathway, SK6 cells were transfected with pGL3-IFN-β-Luc, pRL-TK, Flag-cGAS, Flag-STING, and increasing doses of His-MGF505–6R plasmids, and then subjected to dual luciferase assays. The results showed that MGF505–6R inhibited cGAS-STING-mediated IFN-β promoter activation in SK6 cells in a dose-dependent manner (**Figure 1C**). Furthermore, we also discovered that MGF505–6R reduced cGAS-STING-mediated mRNA levels of IFN-β and the antiviral genes ISG54, ISG56, CXCL10, and TNF-α compared to the empty vector in SK6 cells (**Figure 1D**). To directly visualize the inhibitory effects of MGF505–6R on type I interferon responses, SK6 cells were transfected with His-MGF505–6R or an empty vector and then infected with eGFP-VSV before visualization by fluorescence microscopy. The results indicated that overexpression of MGF505–6R significantly enhanced eGFP-VSV replication in SK6 cells in the presence of poly(dA:dT). The average fluorescence intensity was then analyzed using ImageJ software (**Figure 1E**). Consistently, MGF505–6R also exerted antagonistic effects on the cGAS-STING signaling pathway in HEK-293T cells (**Supplementary Figure 2**). These results demonstrate that MGF505–6R inhibits type I interferon responses by targeting the cGAS-STING signaling pathway.

### 3.2 ASFV MGF505–6R associates with STING to inhibit the cGAS-STING signaling pathway

**Figure 2:**
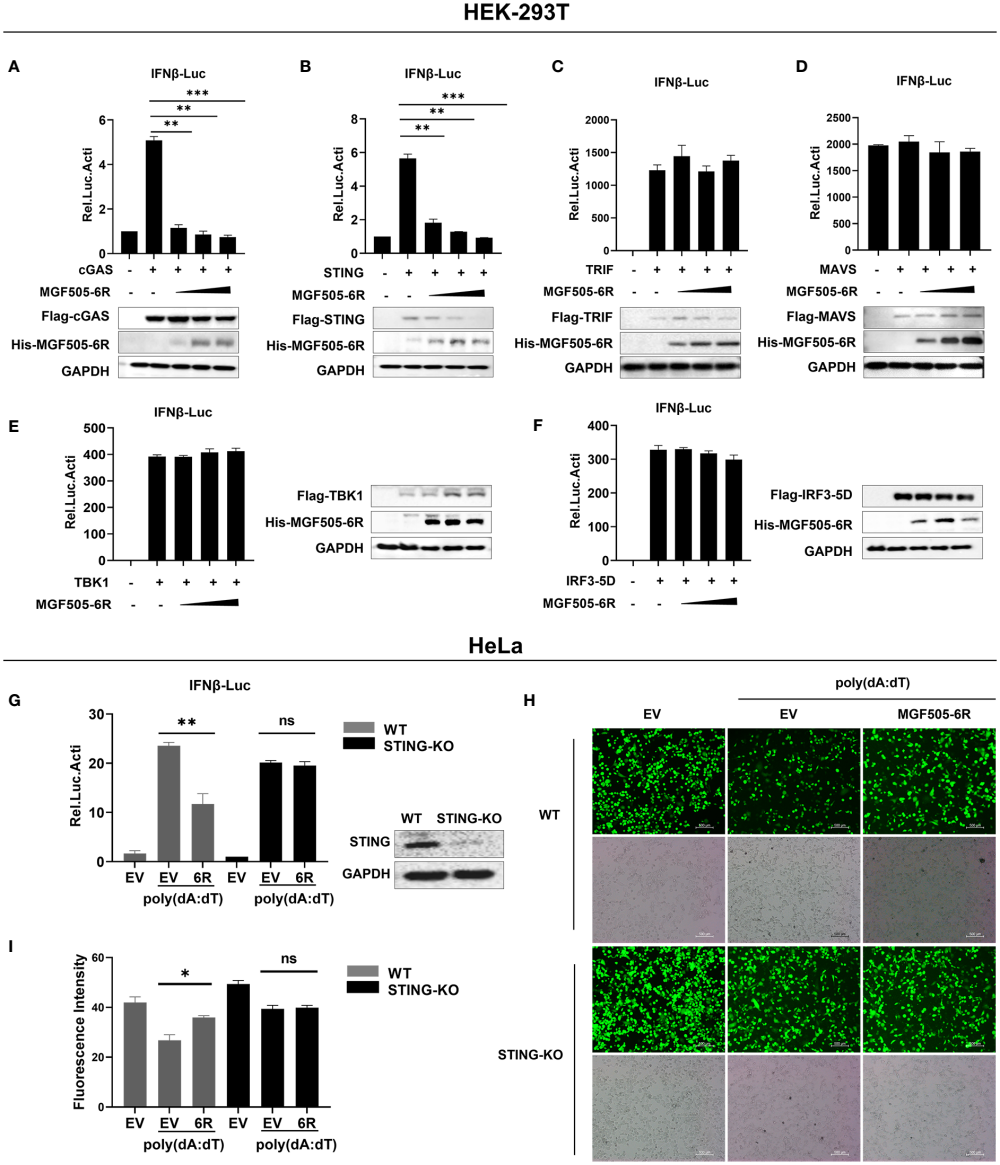
ASFV MGF505–6R associates with STING to inhibit cGAS-STING signaling pathway. **(A–F)** HEK-293T cells were co-transfected with pGL3-IFN-β-Luc, pRL-TK, Flag-cGAS **(A)**, Flag-STING **(B)**, Flag-TRIF **(C)**, Flag-MAVS **(D)**, Flag-TBK1 **(E)**, or Flag-IRF3–5D **(F)**, along with increasing doses of His-MGF505–6R plasmids, and luciferase assays were performed after 24 h transfection. **(G)** His-MGF505–6R or empty vector (EV) together with pGL3-IFN-β-Luc and pRL-TK plasmids were transfected into HeLa WT and STING-KO cells for 24 h. Following that, 1μg/mL poly(dA:dT) was transfected for 12 h before luciferase assays. **(H)** His-MGF505–6R or empty vector (EV) was transfected into HeLa WT and STING-KO cells for 24 h, then 1μg/mL poly(dA:dT) was transfected for 12 h before infection with 0.01 MOI of eGFP-VSV, and eGFP-VSV replication was observed using a fluorescence microscope. **(I)** The average fluorescence intensity was calculated using ImageJ. The data are shown as the mean ± SD; n = 3. *, p < 0.05; **, p < 0.01; ***, p < 0.001; ns, not significant.

We examined signaling molecules of the cGAS-STING, Toll-like receptor, and RIG-I-like receptor signaling pathways that lead to IFN-β production. The results indicated that the ectopic expression of MGF505–6R inhibited the activation of the IFN-β promoter in a dose-dependent manner by suppressing cGAS and STING but not TRIF, MAVS, TBK1, and IRF3 (**Figures 2A–F**). These results demonstrate that STING could be a potential target of MGF505–6R for counteracting the cGAS-STING signaling pathway. To further support this hypothesis, STING-knockdown HeLa cells were generated using shRNA and then subjected to dual luciferase assays. The results showed that MGF505–6R lost its inhibitory effects on poly(dA:dT)-induced IFN-β promoter activation in STING-knockdown HeLa cells (**Figure 2G**). To visualize the impact of MGF505–6R on virus replication in the absence of STING, wild-type and STING-knockdown HeLa cells were infected with eGFP-VSV while overexpressing MGF505–6R and then observed using fluorescence microscopy. The results indicated that MGF505–6R promoted the replication of eGFP-VSV in wild-type HeLa cells but did not affect viral replication in the absence of STING (**Figure 2H**). The average fluorescence intensity was calculated using Image J (**Figure 2I**). Taken together, these results demonstrate that MGF505–6R reduces type I interferon production by targeting STING protein.

### 3.3 ASFV MGF505–6R interacts with STING

**Figure 3:**
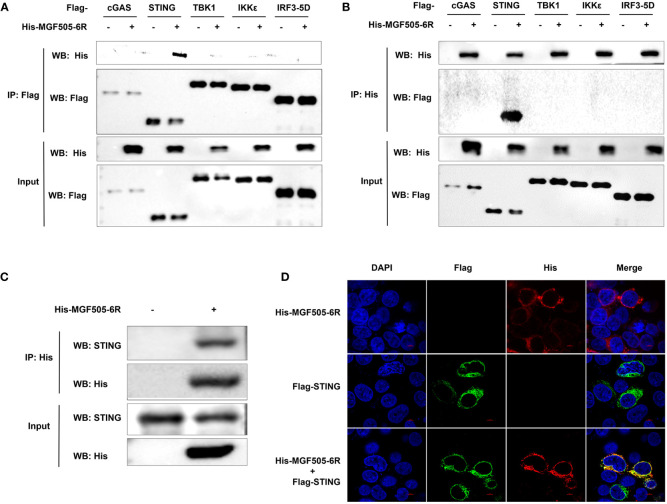
ASFV MGF505–6R interacts with STING. **(A, B)** HEK-293T cells were co-transfected with empty vector or His-MGF505–6R plasmids along with Flag-cGAS, Flag-STING, Flag-TBK1, Flag-IKKϵ, and Flag-IRF3–5D individually, and coimmunoprecipitation and immunoblot analyses were performed with the indicated antibodies after 24 h transfection. **(C)** SK6 cells were transfected with His-MGF505–6R plasmid for 24 h before co-immunoprecipitation and immunoblot analyses with the indicated antibodies. **(D)** HEK-293T cells were transfected with Flag-STING and His-MGF505–6R plasmids, and the colocalization between MGF505–6R and STING was observed using confocal microscopy.

Given that MGF505–6R impairs IFN-β responses by targeting STING, we wanted to investigate whether MGF505–6R directly interacts with STING. Specifically, we screened proteins on the cGAS-STING signaling pathway that were inhibited by MGF505–6R in the IFN-β luciferase assays. The results showed that MGF505–6R only co-precipitated with STING but not with cGAS, TBK1, IKKϵ, or IRF3 using anti-Flag agarose beads (**Figure 3A**) and anti-His agarose beads (**Figure 3B**). Furthermore, we observed interactions between MGF505–6R and endogenous STING in co-IP studies following overexpression of MGF505–6R in SK6 cells (**Figure 3C**). Consistently, colocalization between MGF505–6R and STING was observed in the cytoplasm following the overexpression of MGF505–6R and STING proteins in HEK-293T cells (**Figure 3D**). Together, all the data demonstrate that MGF505–6R interacts with STING to negatively regulate host antiviral immune responses.

### 3.4 ASFV MGF505–6R mediates STING degradation through the autophagy-lysosome pathway

**Figure 4:**
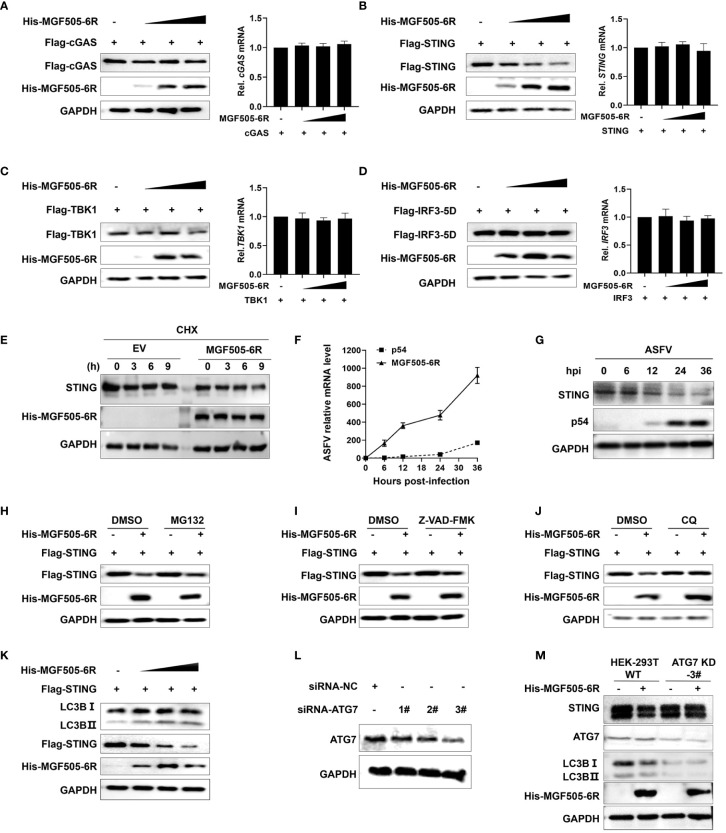
ASFV MGF505–6R mediates STING degradation through the autophagy-lysosome pathway. **(A–D)** HEK-293T cells were transfected with His-MGF505–6R along with Flag-cGAS **(A)**, Flag-STING **(B)**, Flag-TBK1 **(C)**, and Flag-IRF3–5D **(D)** plasmids for 24 h before western blot assays, and the mRNA levels were detected by qRT-PCR. **(E)** HeLa cells transfected with MGF505–6R or empty vector were treated with cycloheximide (CHX) for 0 h, 3 h, 6 h, and 9 h before western blot assays. **(F, G)** Porcine alveolar macrophages (PAMs) infected with ASFV-WT were collected at 0 h, 6 h, 12 h, 24 h, and 36 h post-infection for western blot and qPCR detection. **(H–J)** HEK-293T cells were co-transfected with Flag-STING and His-MGF505–6R for 12 h and then treated with DMSO (negative control), MG132 (10 μM) **(H)**, Z-VAD-FMK (50 μM) **(I)**, or CQ (20 μM) **(J)** for 12 h before western blot detection. **(K)** HEK-293T cells were transfected with Flag-STING and increasing doses of His-MGF505–6R for 24 h before western blot assays with the indicated antibodies. **(L)** HEK-293T cells were transfected with siRNA-negative control (siRNA-NC) and siRNA-targeting ATG7 (siRNA-ATG7) for 12 h before western blot assays. **(M)** HEK-293T cells transfected with siRNA-NC and siRNA-ATG7 were re-transfected with His-MGF505–6R for 24 h before western blot assays.

Considering that MGF505–6R mainly interacts with STING to exert inhibitory effects on type I interferon responses, we wanted to investigate whether MGF505–6R affects the protein levels of STING. HEK-293T cells transfected with Flag-cGAS, Flag-STING, Flag-TBK1, and Flag-IRF3, along with increasing amounts of His-MGF505–6R plasmids, were analyzed using western blot and qRT-PCR. The results show that the overexpression of MGF505–6R significantly reduced the protein level of STING in a dose-dependent manner, while it had no impact on the protein levels of cGAS, TBK1, and IRF3. Meanwhile, the mRNA levels of cGAS, STING, TBK1, and IRF3 remained unaffected subsequent to the overexpression of MGF505–6R (**Figures 4A–D**). We further performed a cycloheximide (CHX) chase experiment to determine how MGF505–6R affects the endogenous STING protein levels. The results showed that overexpression of MGF505–6R significantly decreased STING protein levels compared to HeLa cells transduced with an empty vector after treatment with cycloheximide (CHX), a protein synthesis inhibitor (**Figure 4E**). These demonstrate that MGF505–6R affects the stability of STING. Furthermore, we detected the transcriptional kinetics of MGF505–6R and the STING protein levels in porcine alveolar macrophages (PAMs) during ASFV infection. The results showed that MGF505–6R was transcribed at 6 h post-infection, even earlier than E183L-encoded p54 protein (**Figure 4F**). The amounts of STING obviously decreased at 12 h post-infection, which was reasonably due to MGF505–6R expression (**Figure 4G**). To investigate the protein degradation pathways utilized by MGF505–6R to reduce the protein levels of STING, HEK-293T cells were transfected with His-MGF505–6R and Flag-STING plasmids. Subsequently, the cells were treated with the proteasome inhibitor MG132, the general caspase inhibitor Z-VAD-FMK, and the autophagosome inhibitor CQ individually. The results indicated that the degradation of the STING protein, mediated by MGF505–6R, was effectively rescued by the autophagosome inhibitor CQ but not by the proteasome inhibitor MG132 or the caspase inhibitor Z-VAD-FMK (**Figures 4H–J**). Consistent with this, we observed a dose-dependent increase in the conversion of LC3-I to LC3-II protein with increasing MGF505–6R (**Figure 4K**). To validate the findings, HEK-293T cells with knockdown in the autophagy-related gene (ATG7) were generated using siRNA targeting ATG7 (siRNA-ATG7) (**Figure 4L**). MGF505–6R did not facilitate the transformation of LC3-I to LC3-II and the consequent degradation of STING when ATG7 was knocked down (**Figure 4M**). In summary, these findings demonstrate that MGF505–6R facilitates the degradation of STING through the autophagy-lysosome pathway.

### 3.5 The C-terminal tail (CTT) domain of STING is indispensable for the interaction with MGF505–6R-N (1–260AA)

**Figure 5:**
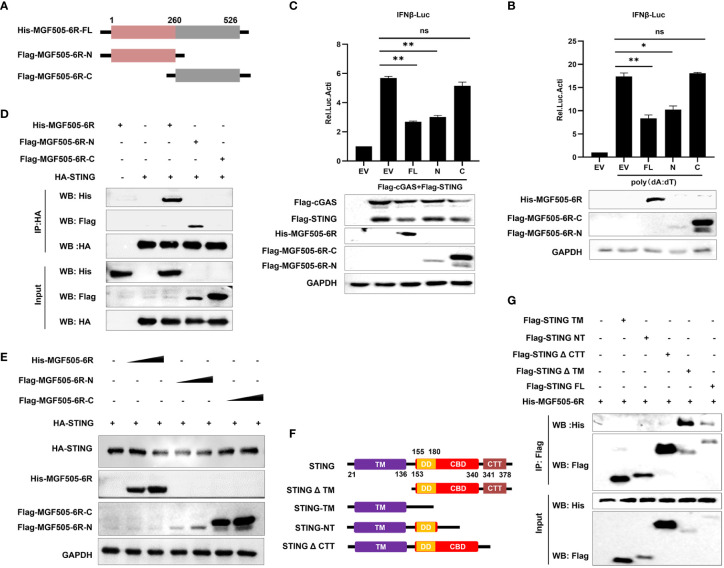
The C-terminal tail (CTT) domain of STING is indispensable for the interaction with MGF505–6R-N (1–260AA). **(A)** The truncations of Flag-MGF505–6R-N and Flag-MGF505–6R-C were truncated as indicated in the pattern diagram and constructed into a pTRIP-CMV vector. **(B)** HEK-293T cells were co-transfected with pGL3-IFN-β-Luc, pRL-TK, along with His-MGF505–6R, Flag-MGF505–6R-N, and Flag-MGF505–6R-C plasmids individually for 24 h, and 1 μg/mL of poly(dA:dT) was transfected for another 12 h before luciferase assays. **(C)** HEK-293T cells were co-transfected with pGL3-IFN-β-Luc, pRL-TK, Flag-cGAS, and Flag-STING, along with His-MGF505–6R, Flag-MGF505–6R-N, and Flag-MGF505–6R-C plasmids individually for 24 h before luciferase assays. **(D)** HEK-293T cells were transfected with HA-STING along with His-MGF505–6R, Flag-MGF505–6R-N, and Flag-MGF505–6R-C individually for 24 h before immunoblot analysis and co-immunoprecipitation with the indicated antibodies. **(E)** HEK-293T cells were co-transfected with HA-STING along with Flag-MGF505–6R-N, Flag-MGF505–6R-C, and His-MGF505–6R plasmids individually for 24 h before western blot detection. **(F)** The truncations of STING were constructed as indicated in the pattern diagram. **(G)** HEK-293T cells were co-transfected with His-MGF505–6R along with Flag-STING, Flag-STING-TM, Flag-STING-NT, Flag-STINGΔCTT, and Flag-STINGΔTM individually for 24 h before co-immunoprecipitation and immunoblot analysis. *, p < 0.05; **, p < 0.01; ns, not significant.

To further investigate which domains of MGF505–6R are involved in the suppression of type I interferon, the gene MGF505–6R was divided into two segments, MGF505–6R-N (1–260AA) and MGF505–6R-C (261–526AA), and subcloned into the pTRIP-CMV vector with a Flag tag, as depicted in **Figure 5A**. Dual luciferase assays were conducted following the ectopic expression of His-MGF505–6R, Flag-MGF505–6R-N, and Flag-MGF505–6R-C plasmids separately in HEK-293T cells. The results indicated that the MGF505–6R-N domain, rather than MGF505–6R-C, was accountable for the inhibition of poly(dA:dT)-stimulated IFN-β promoter activation (**Figure 5B**). Consistently, MGF505–6R-N also exhibited antagonistic effects on cGAS-STING-mediated IFN-β reporter activation (**Figure 5C**). Furthermore, co-IP with anti-HA agarose beads demonstrates that MGF505–6R and MGF505–6R-N, but not MGF505–6R-C, could co-precipitate with STNG (**Figure 5D**). Consistently, the degradation of STING in HEK-293T cells was mediated by MGF505–6R-N rather than MGF505–6R-C (**Figure 5E**). Collectively, the essential domain involved in inhibiting type I IFN responses was MGF505–6R-N. To further explore the domain of STING responsible for its interaction with MGF505–6R, the domains of STING were truncated and constructed as depicted in **Figure 5F**. Co-IP experiments were performed in HEK-293T cells following the ectopic expression of His-MGF505–6R, along with truncation plasmids of Flag-STING-TM, Flag-STING-NT, Flag-STINGΔCTT, Flag-STINGΔTM, and Flag-STING. The results indicated that STING and STINGΔTM, but not STING-TM, STING-NT, or STINGΔCTT truncations, were able to co-precipitate with MGF505–6R when using anti-Flag agarose beads (**Figure 5G**). The data demonstrate that the STING CTT domain plays a crucial role in the interaction with MGF505–6R.

### 3.6 ASFV MGF505–6R protein attenuates K63-linked STING polyubiquitination

**Figure 6:**
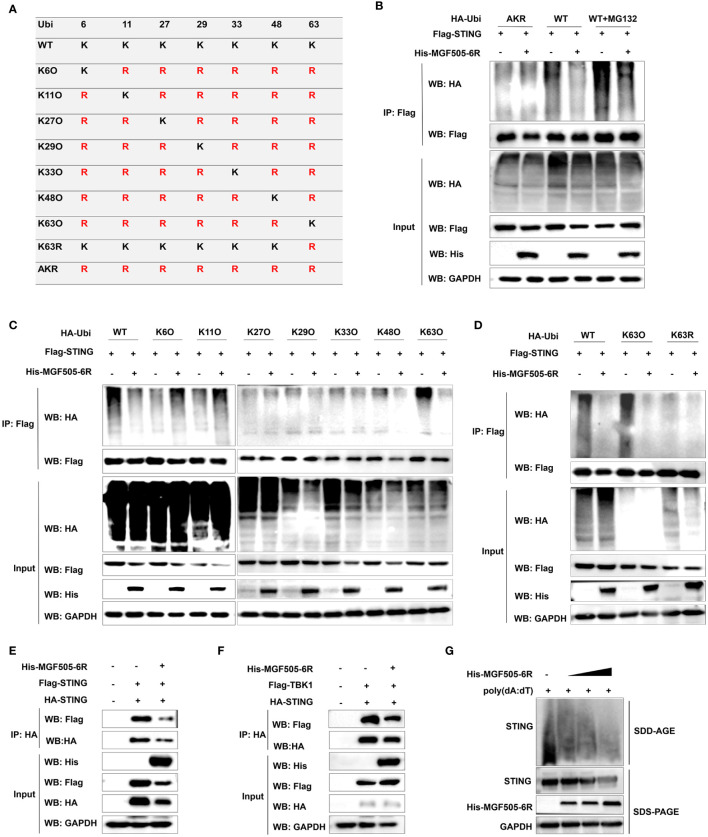
ASFV MGF505–6R attenuates K63-linked STING polyubiquitination. **(A)** HA-tagged ubiquitin and ubiquitin mutants either retaining a single lysine residue (KO) or retaining all but one lysine residue (KR), and mutating all lysine residues to arginine (AKR) were constructed. **(B)** HEK-293T cells were transfected with Flag-STING and HA-Ubi or HA-AKR, along with His-MGF505–6R or empty vector for 24 h, and the cells were either treated with MG132 or not for 12 h before co-immunoprecipitation assays. **(C)** HEK-293T cells were individually transfected with HA-Ubi and mutants K6O, K11O, K27O, K29O, K33O, K48O, and K63O, along with Flag-STING and His-MGF505–6R for 24 h before co-immunoprecipitation assays. **(D)** HEK-293T cells were transfected with Flag-STING and His-MGF505–6R, along with HA-Ubi, mutants K63O and K63R, individually for 24 h before co-immunoprecipitation assays. **(E)** HEK-293T cells were transfected with an empty vector or His-MGF505–6R along with HA-STING and Flag-STING expression plasmids for 24 h before co-immunoprecipitation assays with the indicated antibodies. **(F)** HEK-293T cells were transfected with an empty vector or His-MGF505–6R along with HA-STING and Flag-TBK1 expression plasmids for 24 h before co-immunoprecipitation assays with the indicated antibodies. **(G)** HeLa cells were transfected with increasing doses of His-MGF505–6R for 24 h, and then re-transfected with poly(dA:dT) for 12 h before SDS-PAGE and SDD-AGE detection.

Ubiquitination plays a crucial role in regulating the STING-mediated signaling pathway. To assess the impact of MGF505–6R on STING polyubiquitination, ubiquitin and ubiquitin mutants either retained a single lysine residue (KO) or retained all lysine residues except one (KR), and mutating all lysine residues to arginine (AKR) were constructed (**Figure 6A**). HEK-293T cells transfected with His-MGF505–6R exhibited a significant reduction in STING polyubiquitination, both in the presence and absence of MG132, compared to cells transfected with an empty vector. However, MGF505–6R did not impact the polyubiquitination of STING when all lysine residues were mutated to arginine (AKR) (**Figure 6B**). To examine the polyubiquitin chains linked to STING that were affected by MGF505–6R, co-IP assays were performed on HEK-293T cells transfected with ubiquitin and mutants containing a single lysine residue at positions 6, 11, 27, 29, 33, 48, and 63. The results indicated that MGF505–6R clearly facilitated the elimination of K63-linked ubiquitination from STING while not affecting ubiquitination associated with any other linkage (**Figure 6C**). Moreover, MGF505–6R failed to reduce STING polyubiquitination when the lysine residue at position 63 was substituted with arginine (K63R) (**Figure 6D**). The necessity of K63-linked polyubiquitination for the oligomerization of STING and its recruitment to TBK1 has been documented (30). Our further investigation revealed that MGF505–6R overexpression significantly inhibited the interaction between HA-STING and Flag-STING in HEK-293T cells compared to the empty vector (**Figure 6E**). Consistently, the ectopic expression of MGF505–6R reduced the interaction between STING and TBK1 (**Figure 6F**). Additionally, MGF505–6R overexpression dose-dependently attenuated poly(dA:dT)-induced aggregation of endogenous STING in HeLa cells (**Figure 6G**). In general, MGF505–6R inhibits the K63-linked polyubiquitination of STING, thereby hindering the oligomerization of STING and the recruitment of STING to TBK1.

### 3.7 MGF505–6R impairs the phosphorylation and nuclear translocation of IRF3

**Figure 7:**
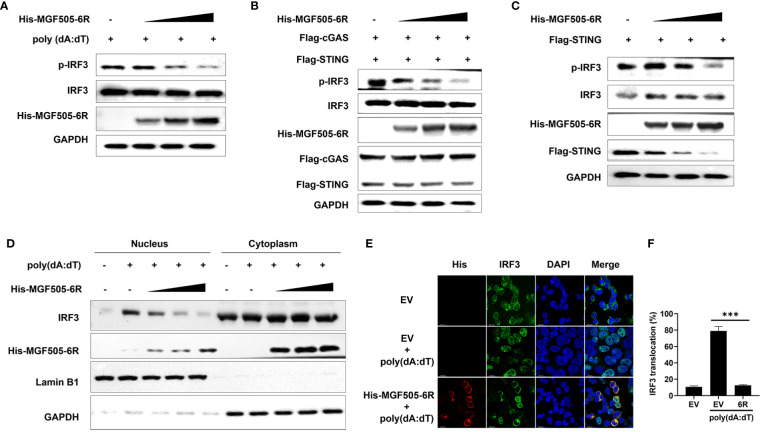
ASFV MGF505–6R impairs the phosphorylation and nuclear translocation of IRF3. **(A)** HEK-293T cells were transfected with an empty vector or increasing doses of His-MGF505–6R expression plasmids for 24 h, and then 1 μg/mL of poly(dA:dT) was transfected for another 12 h before western blot assays. **(B)** HEK-293T cells were transfected with Flag-cGAS, Flag-STING and increasing doses of His-MGF505–6R expression plasmids for 24 h before western blot assays. **(C)** HEK-293T cells were transfected with Flag-STING and increasing doses of His-MGF505–6R expression plasmids for 24 h, and western blot assays were performed with the indicated antibodies. **(D)** HEK-293T cells were transfected with increasing doses of His-MGF505–6R expression plasmids for 24 h. Following that, 1 μg/mL of poly(dA:dT) was transfected for another 12 h, and the subcellular fractions of the nucleus and cytoplasm were subjected to immunoblot analysis. **(E)** HEK-293T cells were transfected with His-MGF505–6R or empty vector for 24 h and then 1 μg/mL of poly(dA:dT) for another 12 **(h)**. After that, the cells were fixed and incubated with the indicated primary antibodies, Alexa Fluor 488- and Alexa Fluor 555-conjugated secondary antibodies for confocal microscopy observation. **(F)** The nuclear IRF3 was analyzed using ImageJ software. ***, p < 0.001.

Upon activation of the cGAS-STING-TBK1 axis, phosphorylation and nuclear translocation of IRF3 are initiated, leading to the generation of type I interferon and subsequent expression of antiviral genes. Our study revealed that overexpression of MGF505–6R notably suppressed the poly(dA:dT)-induced phosphorylation of IRF3 in HEK-293T cells (**Figure 7A**). Consistently, MGF505–6R impaired IRF3 phosphorylation in a dose-dependent manner, which was induced by overexpression of cGAS-STING or STING alone in HEK-293T cells (**Figures 7B, C**). To support the findings, the subcellular fractions of the nucleus and cytoplasm were isolated and underwent immunoblot analysis after transfection with increasing doses of His-MGF505–6R. The results indicated that MGF505–6R reduced the levels of IRF3 in the nucleus in a dose-dependent manner following poly(dA:dT) treatment in HEK-293T cells (**Figure 7D**). Furthermore, we observed an inhibition of poly(dA:dT)-induced nuclear translocation of IRF3 in HEK-293T cells by MGF505–6R overexpression using confocal microscopy (**Figure 7E**). The nuclear IRF3 was quantified using ImageJ (**Figure 7F**). The data collectively demonstrate that MGF505–6R suppresses the phosphorylation and nuclear translocation of IRF3, leading to a subsequent impairment in the production of IFN-β and antiviral genes.

## 4 Discussion

ASFV encodes numerous proteins within the multigene family to evade host antiviral immune responses, thereby playing a pivotal role in viral pathogenicity and imposing tremendous challenges for the development of antiviral vaccines and drugs. In order to facilitate the rational design of live-attenuated vaccines, there has been a growing body of research dedicated to investigating the diverse immune evasion mechanisms utilized by ASFV-encoded proteins. For example, ASFV MGF360–11L interacts with and degrades TBK1 and IRF7 through the cysteine, ubiquitin-proteasome, and autophagy pathways, thereby inhibiting the cGAS-STING signaling pathway (31). Similarly, ASFV MGF360–14L has been documented to facilitate the degradation of the IRF3 protein through ubiquitin-mediated proteolysis, resulting in the suppression of IFN-β (32). Furthermore, ASFV MGF360–9L negatively regulates the IFN-β signaling pathway. This is achieved through its interaction with and subsequent degradation of signal transducer and activator of transcription 1 (STAT1) and STAT2 through apoptosis and ubiquitin-proteasome pathways, respectively (33). More recently, it has been demonstrated that ASFV MGF505–7R inhibits the IFN-β-mediated JAK-STAT signaling pathway by interacting with interferon regulatory factor 9 (IRF9) and impeding the nuclear translocation of ISGF3 (26).

In this study, it was observed that MGF505–6R exhibited antagonistic effects on cGAS-STING-mediated type I interferon responses. Specifically, MGF505–6R facilitated the degradation of the STING protein through the autophagy-lysosome pathway and reduced K63-linked STING polyubiquitination, thereby preventing the phosphorylation and nuclear translocation of IRF3. This ultimately resulted in the downregulation of IFN-β and antiviral genes. All of these studies contribute to the exploration of the intricate immune evasion strategies developed by ASFV, thereby establishing the scientific groundwork for the development of antiviral strategies.

The STING molecule is extensively expressed in various cells and primarily localizes to the endoplasmic reticulum (ER) membrane, where it plays a crucial role in responding to cytosolic dsDNA. STING is activated upon binding to the secondary messenger cGAMP, leading to a conformational change of STING dimers and recruitment of the downstream TBK1 through the CTT domain of STING (19, 34, 35). In this study, we found that MGF505–6R specifically interacts with full-length STING and STINGΔTM, rather than STING-TM, STING-NT, or STINGΔCTT, the truncations without the CTT domain (**Figure 5D**). This reveals that the CTT domain of STING may be essential for its interaction with MGF505–6R. Therefore, we propose that MGF505–6R may competitively interact with the CTT domain of STING to disrupt the dimerization of STING and its recruitment to TBK1. As we hypothesized, we observed that overexpression of MGF505–6R co-precipitated reduced amounts of STING and TBK1 in HEK-293T cells compared to the empty vector (**Figures 6E, F**). Additionally, MGF505–6R reduced the aggregation of endogenous STING in the presence of poly(dA:dT) (**Figure 6G**).

To maintain the immune homeostasis of the host, the stability of STING must be precisely regulated (36). Over-activation of STING brings out excessive inflammation, even leading to autoinflammatory diseases and cancers (37). Post-translational modifications (PTMs) are a crucial strategy for regulating STING stability (37, 38). In the process of virus infection, the stability of the STING protein can be disrupted through virus-mediated degradation by autophagy-lysosome or ubiquitin-proteasome pathways. For example, pseudorabies virus (PRV) tegument protein UL13 regulates STING stability by recruiting the E3 ligase RING-finger protein 5 (RNF5) to promote K27-/K29-linked ubiquitination and degradation of STING (39). ASFV L83L reduces STING protein levels by recruiting Tollip to promote autophagy-lysosomal degradation, thereby negatively regulating the phosphorylation of downstream signaling molecules and interferon production (40). In our study, we observed that overexpression of MGF505–6R led to STING degradation in a dose-dependent manner. The degradation was inhibited by the autophagosome inhibitor CQ and ATG7 knockdown, suggesting that MGF505–6R induces STING degradation through the autophagy-lysosomal pathway. Furthermore, MGF505–6R reduced STING K63-linked polyubiquitination, which is necessary for the transport and activation of STING, thereby impairing signal transduction to downstream molecules and type I interferon production (38). To further corroborate the antagonistic effects of MGF505–6R on host immune defenses, we endeavored to create an ASFV strain deficient in the MGF505–6R gene. Unfortunately, we encountered huge challenges in replicating the ASFV strain when the MGF505–6R gene was deleted from ASFV parental strain. This suggests that MGF505–6R may play a critical role in ASFV virus infection or replication cycle.

## Data availability statement

The original contributions presented in the study are included in the article/**Supplementary Materials**, further inquiries can be directed to the corresponding author/s.

## Ethics statement

Ethical approval was not required for the studies on humans in accordance with the local legislation and institutional requirements because only commercially available established cell lines were used. Ethical approval was not required for the studies on animals in accordance with the local legislation and institutional requirements because only commercially available established cell lines were used.

## Author contributions

MYa: Data curation, Investigation, Validation, Writing – original draft, Writing – review & editing. HC: Investigation, Writing – review & editing. WL: Supervision, Writing – review & editing. ZH: Writing – review & editing, Investigation. ZR: Investigation, Writing – review & editing. MYi: Investigation, Writing – review & editing. LT: Investigation, Writing – review & editing. DH: Investigation, Writing – review & editing. XL: Funding acquisition, Writing – review & editing. PQ: Funding acquisition, Writing – review & editing.
