# Supplementary material for: African swine fever virus MGF505–6R attenuates type I interferon production by targeting STING for degradation
Source: Front Immunol. 2024 May 10;15:1380220. doi: 10.3389/fimmu.2024.1380220 (PMC11116646; doi:10.3389/fimmu.2024.1380220)
Supplement: Supplementary file 1 [file DataSheet_1.pdf]

Supplementary material

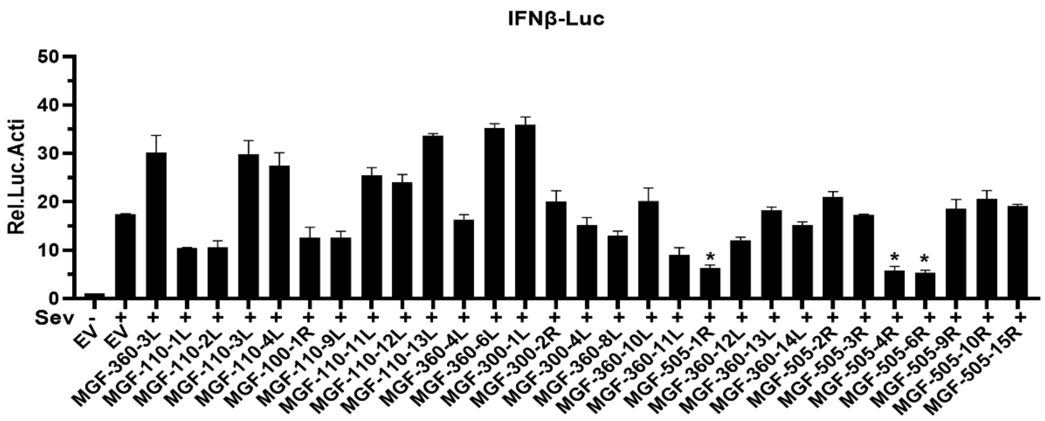

Supplementary Figure 1 MGF505-6R significantly exerts antagonistic effect on SeV-induced IFN-β promoter activation. HEK-293T cells were transfected with pGL3-IFN-β-Luc, pRL-TK, along with expression plasmids of ASFV-encoded proteins for 24 h, and then infected with Sendai virus (SeV) for 8 h before luciferase assays. The data are shown as the mean ± SD; n= 2. *; p < 0.05; **, p < 0.01; ***, p < 0.001.

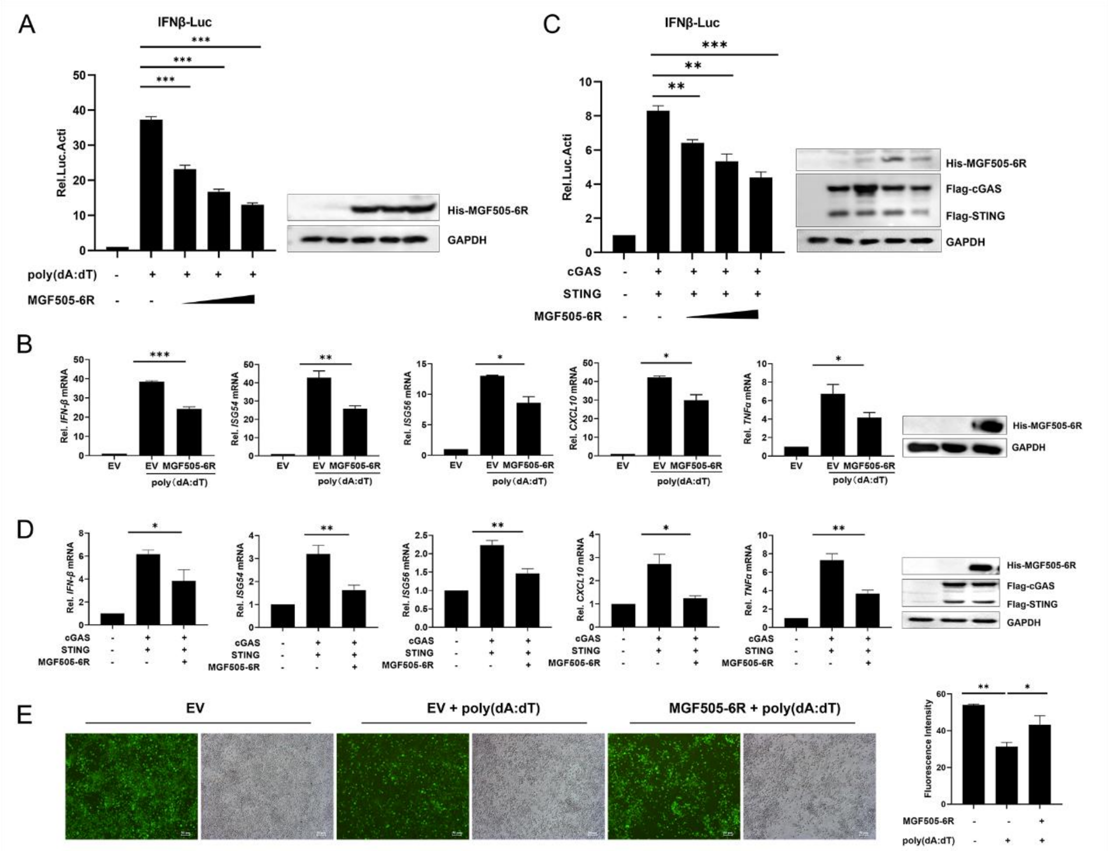

Supplementary Figure 2 ASFV MGF505-6R inhibits cGAS-STING signaling pathway in HEK-

293T cells. (A) HEK-293T cells were transfected with pGL3-IFN-β-Luc (0.1 μg), pRL-TK (0.05 μg), and increasing doses of His-MGF505-6R expression plasmids for 24 h. Following that, 1 μg/mL of poly(dA:dT) was transfected for another 12 h before luciferase assays. (B) HEK-293T cells were transfected with His-MGF505-6R for 24 h and then retransfected with 1 μg/mL of poly(dA:dT) for 12 h before the detection of IFN-β, ISG54, ISG56, CXCL10, and TNFα mRNA levels. (C) HEK-293T cells were transfected with pGL3-IFN-β-Luc, pRL-TK, Flag-cGAS, and Flag-STING, along with increasing doses of His-MGF505-6R expression plasmid for 24 h before luciferase assays. (D) HEK-293T cells were transfected with Flag-cGAS, Flag-STING and His-MGF505-6R for 24 h before the detection of IFN-β, ISG54, ISG56, CXCL10 and TNFα mRNA levels. (E) HEK-293T cells transfected with an empty vector (EV) or His-MGF505-6R were retransfected with poly(dA:dT) for 12 h, and then infected with 0.01 MOI of eGFP-VSV for 12 h before microscope observation. The average fluorescence intensity was analyzed on ImageJ software. The data are shown as the mean ± SD; n = 3. *, $p < 0.05$; **, $p < 0.01$; ***p, < 0.001; ns, not significant.

Supplementary Table 1 Primers used for qRT-PCR in this study.

Primers	Sequence (5' to 3')
Human IFN-β-forward	TCTTTCCATGAGCTACAACTTGCT
Human IFN-β-reverse	GCAGTATTCAAGCCTCCCATTC
Human ISG54-forward	AATAGGACACGCTGTGGCTC
Human ISG54-reverse	GGTGGATGGCCTTGTCTTCA
Human ISG56-forward	GAAGGATGGGCCTTGCTGAA
Human ISG56-reverse	CAGGCGATAGGCAGAGATCG
Human CXCL10-forward	GAACTGTACGCTGTACCTGC
Human CXCL10-reverse	AACACGTGGACAAAATTGGCT
Human TNF-α-forward	TCTTCTCGAACCCCGAGTGA
Human TNF-α-reverse	TATCTCTCAGCTCCACGCCA
Human GAPDH-forward	AAATTCCATGGCACCGTCAA
Human GAPDH-reverse	TGGTTCACACCCATGACGAA
Pig IFN-β-forward	CACTGGCTGGAATGAAACCG
Pig IFN-β-reverse	AATGGTCATGTCTCCCCTGG
Pig ISG54-forward	CTGGCAAAGAGCCCTAAGGA
Pig ISG54-reverse	CTCAGAGGGTCAATGGAATTCC
Pig ISG56-forward	TTAGAAAACAGGGTCTTGGAGGAG
Pig ISG56-reverse	CGTAAGGTAATACAGCCAGGCATA
Pig CXCL10-forward	CCCACATGTTGAGATCATTGC
Pig CXCL10-reverse	CATCCTTATCAGTAGTGCCG
Pig TNF-α-forward	GCCCAAGGACTCAGATCATC
Pig TNF-α-reverse	GGCATTGGCATACCCACTCT
Pig GAPDH-forward	ACATGGCCTCCAAGGAGTAAGA
Pig GAPDH-reverse	GATCGAGTTGGGGCTGTGACT
Human cGAS-forward	GGAGCCCTGCTGTAACACTT
Human cGAS-reverse	GTGAGAGAAGGATAGCCGCC

Human STING-forward	TGGCATCAAGGATCGGGTTT
Human STING-reverse	GTCATCTGCAGGTTCCGCTG
Human TBK1-forward	TTGCAGTCTTTCTCGGGGTC
Human TBK1-reverse	GCGTCGCCCTTCGTAGATAA
Human IRF3-forward	AGGATGCACAGCAGGAGGAT
Human IRF3-reverse	TGTCTGGCTGGGAAAAGTCC
ASFV p54-forward	CACTACACCAAGCTTCTTCTCCA
ASFV p54-reverse	TCTTCCTCCTCAATAGCAGC
MGF505-6R-forward	ATTAACCAATGGAGTTCGCATG
MGF505-6R-reverse	ATAAGGCTCTATGTATATTCCCACC

Supplementary Table 2 Sequences of siRNA targeting ATG7

Primers	Sequence (5’ to 3’)
siRNA-ATG7 1# sense	GCCAGUGGGUUUGGAUCAATT
siRNA-ATG7 1# antisense	UUGAUCCAAACCCACUGGCTT
siRNA-ATG7 2# sense	GCGUGAGACACAUCACAUUTT
siRNA-ATG7 2# antisense	AAUGUGAUGUGUCUCACGCTT
siRNA-ATG7 3# sense	CCAACAUCCCUGGUUACAATT
siRNA-ATG7 3# antisense	UUGUAACCAGGGAUGUUGGTT
